# Enhancing survival prediction for COVID-19 in diabetic patients in Mexico: integrating RMST, propensity score matching, and ensemble machine learning

**DOI:** 10.3389/fendo.2025.1725251

**Published:** 2026-01-12

**Authors:** Mariano Vargas-Santiago, Diana A. León-Velasco, Raúl Monroy, Sergio Quezada-García

**Affiliations:** 1 Secretaría de Ciencia, Humanidades, Tecnologico e Innovación (SECIHTI-IXM), Ciudad de México, Mexico; 2 Universidad Autónoma Metropolitana, Unidad Azcapotzalco, Departamento de Sistemas, Ciudad de México, Mexico; 3 Tecnologico de Monterrey, School of Engineering and Science, Atizapán, Estado de México, Mexico; 4 Facultad de Ingeniería, Universidad Nacional Autónoma de México, Ciudad de México, Mexico

**Keywords:** COVID-19, diabetes, ensemble models, machine learning, propensity score matching, RMST, survival prediction, viral infections

## Abstract

**Background:**

This study evaluates the survival impact of diabetes on hospitalized COVID-19 patients in Mexico by combining traditional survival methods (Restricted Mean Survival Time, RMST) with machine learning (ML) prediction. The goal is to understand how diabetes and associated comorbidities affect short-term survival and to develop accurate, interpretable models that support data-driven decision-making.

**Methods:**

A national dataset of over one million COVID-19 cases was analyzed. Diabetic and non-diabetic cohorts were matched using propensity scores based on key covariates (e.g., age, gender, and comorbidities). RMST differences were estimated using survival curves and statistical testing. Separately, machine learning models (Random Forest (RF) and Variational Deep Neural Network (VDNN)) were trained to predict individual RMST values, and SHapley Additive exPlanations (SHAP) were used for model interpretability.

**Results:**

The RMST for diabetic patients was lower than that for non-diabetic patients, with a difference of 2.32 days (*p* = 0.0583) after matching. Predictive models achieved strong internal validity (*R*^2^ > 0.60). SHAP analysis revealed obesity, smoking, and hypertension as the top predictors and suggested that temporal variables and comorbidities played a central role in short-term survival.

**Conclusion:**

Combining survival analysis with machine learning provides both inferential and predictive insights into the mortality risk of diabetic COVID-19 patients. More importantly, results show that traditional survival analyzes with modern machine learning yields accurate and interpretable predictions that can support personalized interventions tailored to patients with COVID-19 and comorbid diabetes: such as prioritizing early clinical monitoring, individualized treatment plans, or risk-informed hospital admission decisions, and guide a more efficient allocation of healthcare resources.

## 1 Introduction

COVID-19, caused by the SARS-CoV-2 virus, emerged in Wuhan, China, in December 2019 and rapidly spread worldwide Shi et al. (1–3). In Mexico, the first case was reported on February 28, 2020, and since then, more than 7 million confirmed cases and over 300,000 deaths have been documented (4, 5). Although approximately 200 million vaccine doses have been administered, covering nearly three quarters of the population, the country continues to face a high burden of severe COVID-19 due to the combined effects of population aging and the high prevalence of chronic comorbidities associated with adverse outcomes (6, 7).

Mexico has one of the highest prevalences of overweight and obesity in Latin America, affecting more than 10% of the population, and an estimated 13 million people live with diabetes, representing a 7% increase since 2006 (8). Diabetes is associated with immune dysregulation, chronic inflammation, and frequent coexistence with hypertension and other cardiometabolic conditions, all of which increase susceptibility to respiratory infections, including COVID-19 (9–11). Consistent evidence from multiple countries indicates that individuals with diabetes are at higher risk of severe COVID-19, intensive care admission, multiorgan failure, and death, with reported mortality rates in diabetic COVID-19 cohorts ranging from 11% to 33% (12–18).

Several studies have examined the interplay between diabetes, other comorbidities, and COVID-19 outcomes using retrospective cohorts and survival-analysis methods, most commonly Cox proportional hazards models, to identify prognostic factors such as age, sex, and underlying chronic diseases (12–17). However, many of these analyzes have limited generalizability to middle-income settings such as Mexico and may not fully capture complex, nonlinear relationships between comorbidity patterns, temporal factors, and survival. Moreover, there is comparatively little work integrating advanced machine learning techniques with survival metrics such as the restricted mean survival time (RMST) to improve short-term survival prediction in large, real-world datasets.

Recent studies have explored the intersection of diabetes and COVID-19 using various analytical approaches. Notably, (19) applied machine learning techniques, such as Principal Component Analysis (PCA) and Linear Discriminant Analysis (LDA), to identify clinical and metabolic factors associated with susceptibility to COVID-19 among patients with type 2 diabetes. Their findings highlighted the relevance of indicators such as HDL-C, eGFR, and triglyceride levels in modulating the risk of infection. While that study provides valuable insights into susceptibility patterns, our work addresses a complementary and equally critical dimension: the prediction of mortality risk in diabetic patients hospitalized with COVID-19. By integrating RMST analysis with advanced predictive models—namely, Random Forest Regressors (RFRs) and variational deep neural networks (VDNNs)—our study aims to improve survival estimation and inform clinical decision-making. This integrative approach provides a more comprehensive understanding of disease progression and supports the development of targeted and effective interventions for high-risk populations in resource-constrained settings, such as Mexico.

In this study, we address these gaps by analyzing nationwide Mexican data on hospitalized adult patients with laboratory-confirmed COVID-19, focusing on the impact of preexisting diabetes on mortality. We combine traditional survival analysis tools (Kaplan–Meier curves, Cox proportional hazards models, and RMST) with machine learning models, including Random Forests (RF) and VDNNs, applied to RMST estimates for predefined comorbidity–time strata. This hybrid framework allows us to quantify group-level differences in survival between patients with and without diabetes and to explore how comorbidities and temporal factors contribute to predicted short-term survival in the Mexican context.

This study makes three main contributions. First, we integrate traditional survival analysis and modern machine learning by combining RMST, propensity score matching, and ensemble models to enhance interpretability and predictive performance for COVID-19 survival outcomes among diabetic patients. Second, we conduct a large-scale population analysis in Mexico, using a national cohort of more than one million hospitalized cases to provide one of the most comprehensive evaluations of diabetes-related survival disparities in Latin America. Third, we apply explainable artificial intelligence techniques, specifically SHapley Additive exPlanations (SHAP), to interpret model behavior and identify the most influential comorbidities and temporal factors affecting survival in the Mexican population. Together, these elements define a data-driven framework for personalized health decisions that can be extended beyond diabetes to other chronic conditions and viral or respiratory infections, such as influenza, respiratory syncytial virus (RSV), chronic obstructive pulmonary disease (COPD) exacerbations, and other metabolic–infectious comorbidity profiles. These conditions share pathophysiological features with COVID-19–diabetes interactions (e.g., systemic inflammation, impaired immune responses, metabolic dysregulation), making the framework well suited for developing early-warning systems, risk stratification tools, and resource-allocation strategies across diverse clinical and epidemiological contexts.

From a methodological perspective, traditional survival analysis and modern machine learning address related but distinct questions. Classical survival tools such as Kaplan–Meier estimators, Cox proportional hazards models, and RMST are primarily designed for population-level inference, quantifying effect sizes and differences in survival between predefined groups. In contrast, machine learning algorithms are optimized for prediction, focusing on how well future outcomes can be estimated for new observations given a set of covariates. In this study, we explicitly treat these approaches as complementary: survival models are used to characterize the average impact of diabetes on COVID-19 mortality, whereas machine learning models approximate expected short-term survival for specific comorbidity profiles based on RMST estimates.

The remainder of this manuscript is organized as follows: Section 2 describes the dataset, preprocessing steps, and statistical methods, including RMST computation and propensity score matching. Section 3 presents the proposed machine learning framework, detailing the RF, VDNN, and ensemble configurations, and reports the experimental results, including survival comparisons, model performance metrics, and SHAP-based interpretability. Section 4 analyzes our findings considering the survival and machine-learning results, highlights the complementary roles of Cox, KM, and RMST analyses, and examines the biological, public-health, and methodological implications of our work, including its main strengths and limitations. Finally, Section 5 concludes the paper by summarizing key findings, discussing implications for healthcare policy, and proposing future research directions.

## 2 Methods

This retrospective observational study used data from the Mexican Federal Government. The database is publicly available and has been validated by the Epidemiological Surveillance System for Viral Respiratory Diseases of the Mexican Ministry of Health. Ethical approval for the use of these anonymized data was obtained from the ethics committees of the Ministry of Health.

### 2.1 Dataset

We analyzed the COVID-19 Mexican Patients Dataset to characterize demographic, clinical, and outcome patterns in the Mexican population during the COVID-19 pandemic. The dataset was compiled and released by the Mexican Ministry of Health and contains records from 475 Viral Respiratory Disease Monitoring Units and affiliated medical facilities. It includes individuals with a positive COVID-19 test who required hospitalization. For the present study, we considered the period from January 1, 2023, to August 8, 2023, yielding 1,021,380 hospitalized patients with complete information on mortality outcomes.

### 2.2 Determination of COVID-19

COVID-19 diagnosis was based on detection of SARS-CoV-2 antigen using nasal swab testing performed at surveillance and healthcare facilities under the jurisdiction of the Mexican Government. Positive COVID-19 status could be confirmed by one of three procedures routinely applied in the national surveillance system: (i) clinical–epidemiological association, (ii) validation by an expert committee, or (iii) positive antigen test. A negative result indicated absence of detectable SARS-CoV-2 antigen in the tested sample.

### 2.3 Statistical analysis

Demographic and diabetes-related characteristics of SARS-CoV-2–positive individuals were summarized using descriptive statistics. Comparisons between groups were performed using Student’s *t*-tests for continuous variables and $\chi^{2}$ tests for categorical variables. The primary endpoint was survival time, defined as the interval from onset of COVID-19 symptoms to death, with censoring at the last date of follow-up for hospitalized adult patients. Survival functions were estimated using Kaplan–Meier methods, and differences between patients with and without diabetes were evaluated with the log-rank test. Cox proportional hazards models were fitted to estimate hazard ratios and their 95% confidence intervals (CIs) for the association between diabetes and mortality. All statistical tests were two-sided, and *p*-values< 0.05 were considered statistically significant.

In addition to the overall survival analysis, we calculated the RMST for both diabetic and non-diabetic adult COVID-19 patients admitted to hospitals, following propensity score matching to mitigate the impact of confounding variables. RMST was defined as the area under the survival curve up to a pre-specified time horizon *τ* and therefore represents an integral summary of group-level survival rather than an individual patient-level score. To obtain these RMST estimates, we fitted parametric survival models to the matched patient-level data for each exposure group and numerically integrated the corresponding survival functions from time zero to *τ*. The resulting group-level RMST values, together with their 95% confidence intervals and *p*-values, were used to quantify differences in short-term survival between patients with and without diabetes and to construct the prediction targets for the machine learning models described in Section 2.4.

### 2.4 Machine learning approach

Following the RMST analysis, our study implements an extensive machine learning strategy to delve deeper into the dataset. The machine learning component is designed to operate on aggregated survival summaries and comorbidity profiles, rather than on raw patient-level time-to-event data. Importantly, RMST is not defined at the level of a single patient, but as an integral summary of the survival curve for a group of individuals over a fixed time horizon. To use RMST as a prediction target, we first fitted the parametric survival models described in Section 2.3 to the patient-level data and obtained RMST estimates for predefined comorbidity and temporal strata (for example, diabetes with obesity, diabetes with hypertension, etc.). Each row in the machine learning dataset therefore corresponds to one such stratum and contains its estimated RMST together with associated summary statistics (confidence interval bounds, *p*-value) and descriptive covariates (disease category, temporal indicators, and other clinical factors). When we refer to “RMST prediction” in the results, we thus mean the prediction of the expected group-level RMST for a new combination of covariates, rather than the computation of an individual RMST score for a single patient. The machine learning component is therefore not intended to replace the inferential survival models described above, but to complement them by addressing a different question. While Cox models and RMST summaries quantify how diabetes and other covariates affect group-level survival and its uncertainty, the machine learning regressors operate on RMST estimates computed for predefined comorbidity–time strata and are used to capture complex, potentially non-linear relationships between covariates and short-term survival. In this way, the inferential survival analysis provides effect-size estimation and hypothesis testing, whereas the machine learning models focus on predictive performance for new risk profiles.

For a detailed description of the RMST computation and the construction of the aggregated machine learning dataset, see Sections 2.3 and 2.4, as well as the data-transformation pipeline in Appendix **Figure 1**.

**Figure 1:**
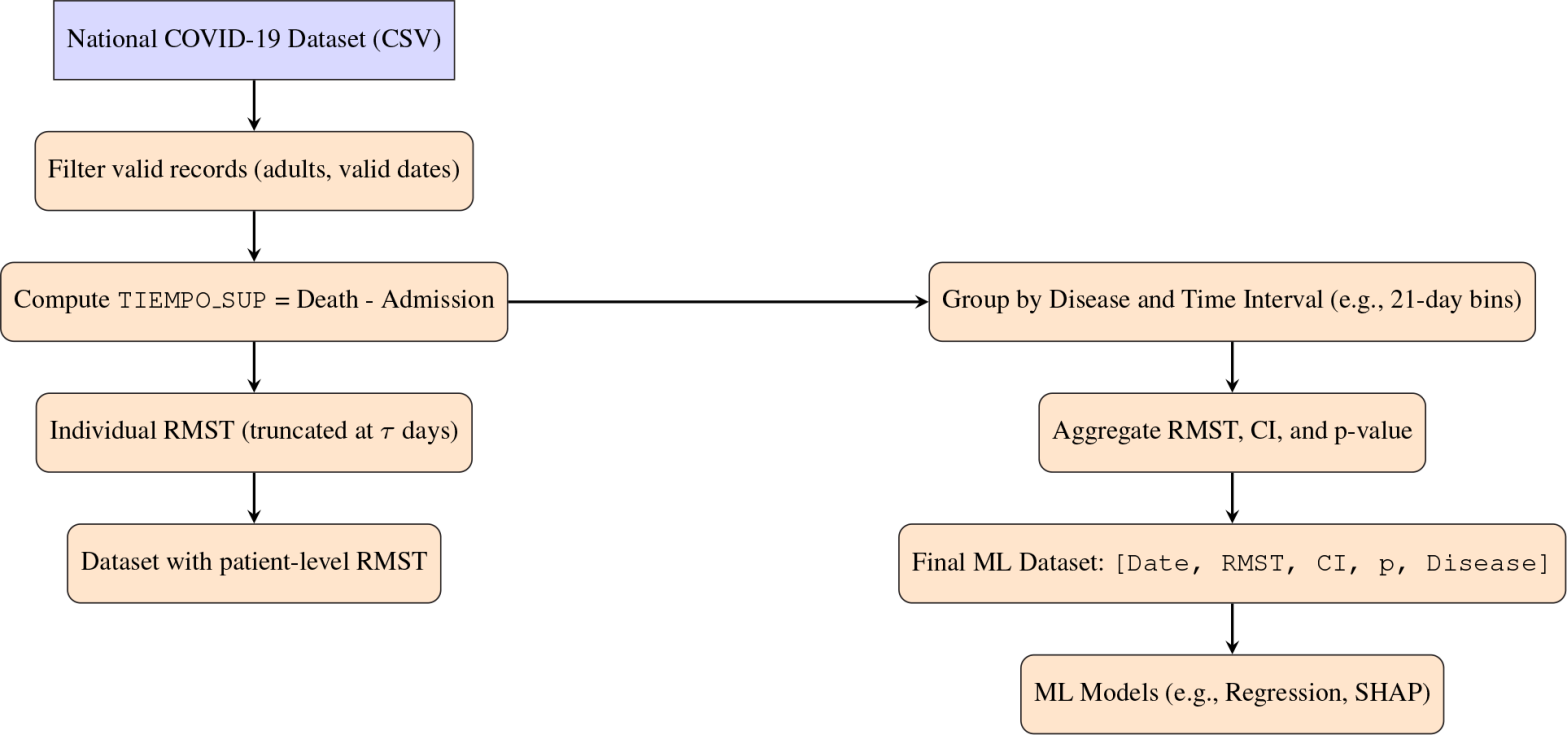
illustrates the two-phase data transformation workflow used in our study. In the first phase, we calculated patient-level Restricted Mean Survival Time (RMST) using the time from admission to death or censoring. In the second phase, we aggregated these results by time intervals (e.g., every 21 days) and stratified them by comorbidities (e.g., smoking, hypertension). This process produced a structured dataset with RMST values per disease and time bin, enabling regression and SHAP-based interpretability analysis. Data transformation pipeline: from raw patient-level records to a structured dataset suitable for machine learning. Patient-level RMST is computed based on time-to-death or censoring, and then aggregated by disease and time interval to model survival patterns over time.

#### 2.4.1 Data preprocessing

The analysis dataset was obtained from combined_covid_comorbidities.csv. Missing values in the RMST variable were imputed using the column mean. We derived an additional feature, the confidence-interval range, defined as the difference between the upper and lower confidence interval bounds. The data were partitioned into predictors (*X*) and target (*y*, RMST), and further split into training and test sets. Numerical predictors were standardized to zero mean and unit variance, and categorical predictors were one-hot encoded using a column-wise preprocessing pipeline based on a ColumnTransformer.

#### 2.4.2 Model training

Two regression models were fitted to predict RMST. First, a Random Forest Regressor (RFR) was used as a nonparametric ensemble of decision trees capable of capturing nonlinear effects and interactions. Second, a fully connected deep neural network (VDNN) was implemented in TensorFlow, with two hidden layers comprising 128 and 64 units, respectively, with ReLU activations, and a single linear output neuron. The VDNN was optimized with the Adam algorithm using mean squared error (MSE) as the loss function. Both models were trained on the preprocessed training set and evaluated on the held-out test set.

#### 2.4.3 Ensemble technique

To further improve predictive performance, we implemented a stacking ensemble. Predictions from the trained RFR and VDNN models on the training data were used as inputs to a meta-learner, specified as a linear regression model. This meta-model was trained to combine the base-model predictions, and its performance was assessed on the test set using MSE, allowing comparison with the individual base models.

#### 2.4.4 Propensity score matching and confounding control

Propensity score matching (PSM) was used to reduce confounding between diabetes and related comorbidities. Propensity scores were estimated with a logistic regression model including age, sex, obesity, hypertension, immunosuppression, chronic obstructive pulmonary disease (COPD), smoking status, and pneumonia as covariates. Patients with diabetes were matched 1:1 to patients without diabetes using nearest-neighbor matching without replacement and a caliper of 0.2 standard deviations of the logit of the propensity score. Covariate balance before and after matching was evaluated using standardized mean differences (SMDs), with |SMD|< 0.1 considered acceptable, and by visual inspection of Love plots. RMST was then estimated in the matched cohort using the same restricted time window and stratification as in the main analysis.

## 3 Results

### 3.1 Clinical characteristics and survival analysis

**Table 1** summarizes the demographic characteristics of the 1,021,380 hospitalized adult COVID-19 patients included in the analysis. Overall, 7.9% were classified as diabetic, 92.0% as non-diabetic, and 0.1% had missing diabetes status. Females represented 58.6% of the cohort and males 41.4%. The mean age was 38.4 years (standard deviation 19.5). The distribution of diabetes by sex was similar to that of the overall population, with 38% of diabetic patients being male and 62% female (**Table 2**).

**Table 1:** Patient distribution by gender among diabetic and nondiabetic groups.

Gender	Diabetic	Nondiabetic	No information	Total individuals
Male	30,533	391,404	407	422,344 (41.4%)
Female	49,813	548,631	592	599,036 (58.6%)
Total Individuals	80,346 (7.9%)	940,034 (92%)	999 (0.1%)	1,021,380 (100%)

**Table 2:** Gender proportions in diabetic and nondiabetic populations.

Gender	Diabetic	% Individuals diabetic	Nondiabetic	% Individuals nondiabetic
Male	30,533	38%	391,404	42%
Female	49,813	62%	548,631	58%
Total	80,346	100%	940,035	100%

**Table 3** shows the prevalence of major comorbidities in diabetic and non-diabetic patients. Hypertension and obesity were markedly more frequent among diabetic patients (55.8% and 20.0%, respectively) than among non-diabetic patients (7.2% and 6.6%). Smoking was also slightly more common in diabetic than in non-diabetic patients (6.2% vs 4.0%). At the population level, 7.87% of subjects had diabetes, 10.98% had hypertension, 7.64% were obese, and 4.09% reported smoking (**Table 4**), underscoring the clustering of cardiometabolic comorbidities among individuals with diabetes.

**Table 3:** Distribution of demographic and clinical covariates in diabetic and nondiabetic patients.

Covariates	Diabetic	% Individuals diabetic	Nondiabetic	% Individuals nondiabetic
Native	80,199	99.8%	935,950	99.6%
Hypertension	44,851	55.8%	67,248	7.2%
Obesity	15,989	20.0%	61,978	6.6%
Smoking	5,007	6.2%	36,773	4.0%
Pneumonia	6,241	7.8%	22,061	2.3%
ICU	508	0.6%	2,181	0.2%
Intubation	809	1.0%	2,607	0.3%
Death	2,198	2.7%	4,371	0.5%

**Table 4:** Distribution of demographic and clinical covariates across all individuals.

Covariates	% Diabetic	% Nondiabetic	Total individuals
Native	7.85%	91.64%	99.49%
Diabetes	7.87%	0%	7.87%
Hypertension	4.39%	6.58%	10.98%
Obesity	1.57%	6.07%	7.64%
Smoking	0.49%	3.60%	4.09%
Pneumonia	0.61%	2.16%	2.77%
ICU	0.05%	0.21%	0.26%
Intubation	0.08%	0.26%	0.33%
Death	0.22%	0.43%	0.64%

**Figure 2:**
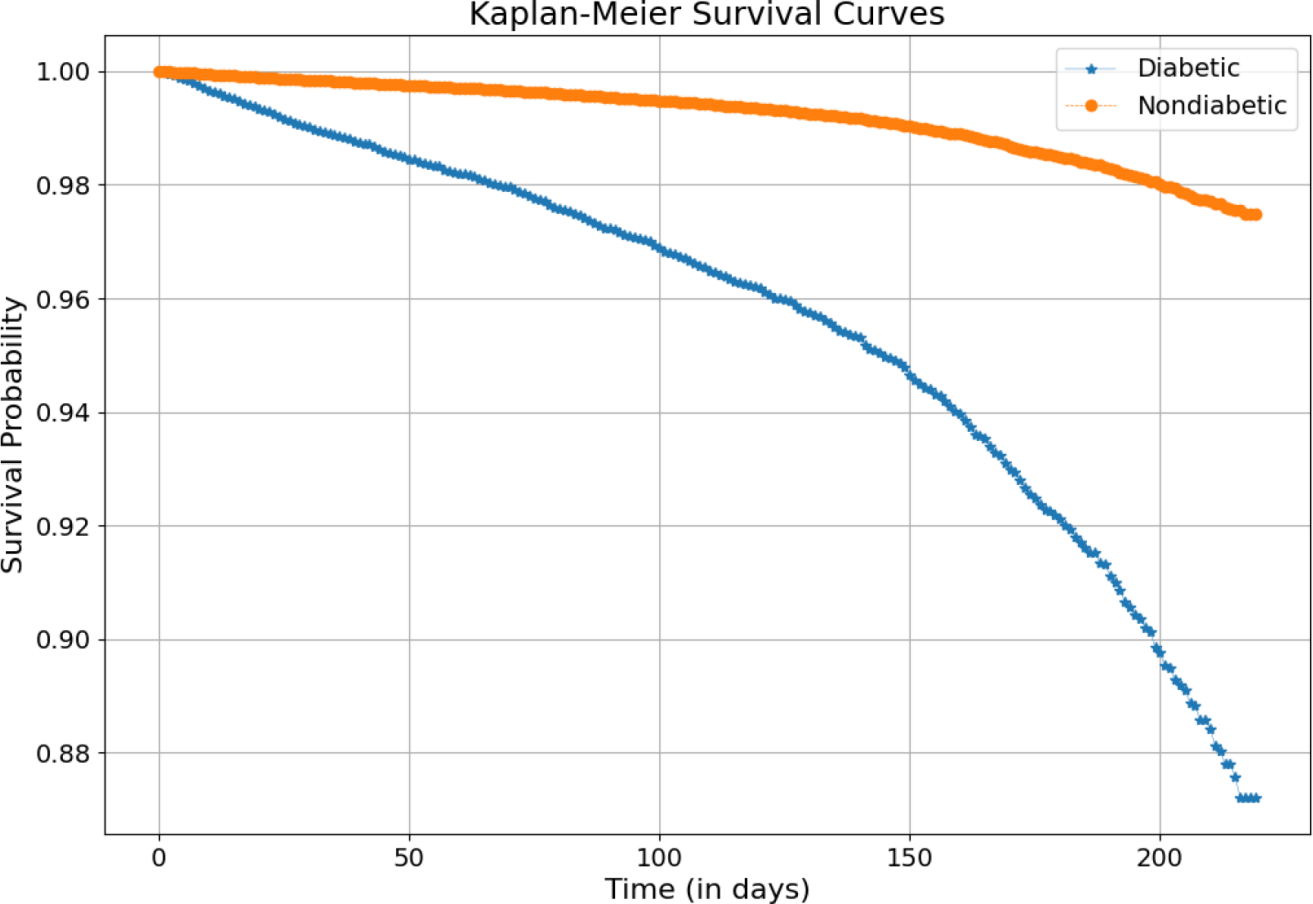
Multivariable Kaplan–Meier survival plots comparing diabetic and nondiabetic patients. The survival probability declines more steeply for the diabetic group, indicating a higher cumulative mortality over time.

Kaplan–Meier curves comparing survival between patients with and without diabetes are displayed in **Figure 2**. Among the 1,021,380 patients, 6,581 deaths were recorded during follow-up. Survival probabilities were consistently lower in the diabetic group, and the log-rank test indicated a statistically significant difference in survival between diabetic and non-diabetic patients (*p<* 0.01). The curves did not cross over time, supporting the proportional hazards assumption.

**Figure 3:**
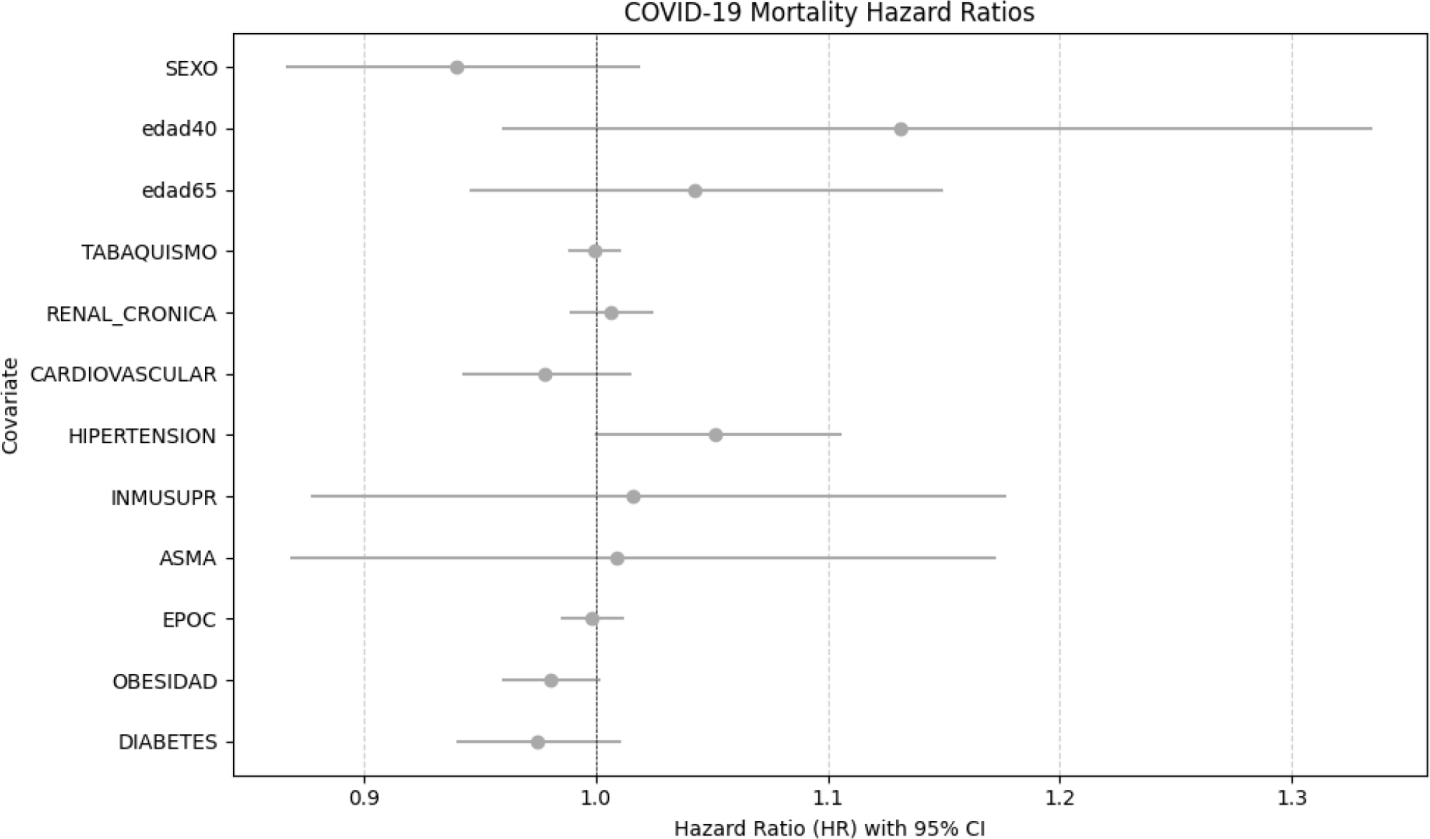
COVID-19 cox mortality hazard ratios for key comorbidities and demographic covariates. Error bars represent 95% confidence intervals. A hazard ratio above 1 indicates increased mortality risk, while below 1 indicates reduced risk.

The multivariable Cox proportional hazards model (**Figure 3**) was used to assess the independent association of diabetes and other covariates with mortality. Diabetes showed a hazard ratio (HR) of 0.975 (95% CI not significantly different from 1, *p* = 0.167), indicating no statistically significant independent effect after adjustment for covariates. Obesity was associated with a small, borderline reduction in mortality risk (HR 0.981, *p* = 0.080), whereas hypertension showed a modest increase in risk (HR 1.051, *p* = 0.051). Age greater than 65 years, immunosuppression, and chronic renal disease were associated with clearly elevated hazards, while asthma and several other comorbidities had CIs crossing unity, indicating a lack of statistically significant association with mortality.

**Figure 4:**
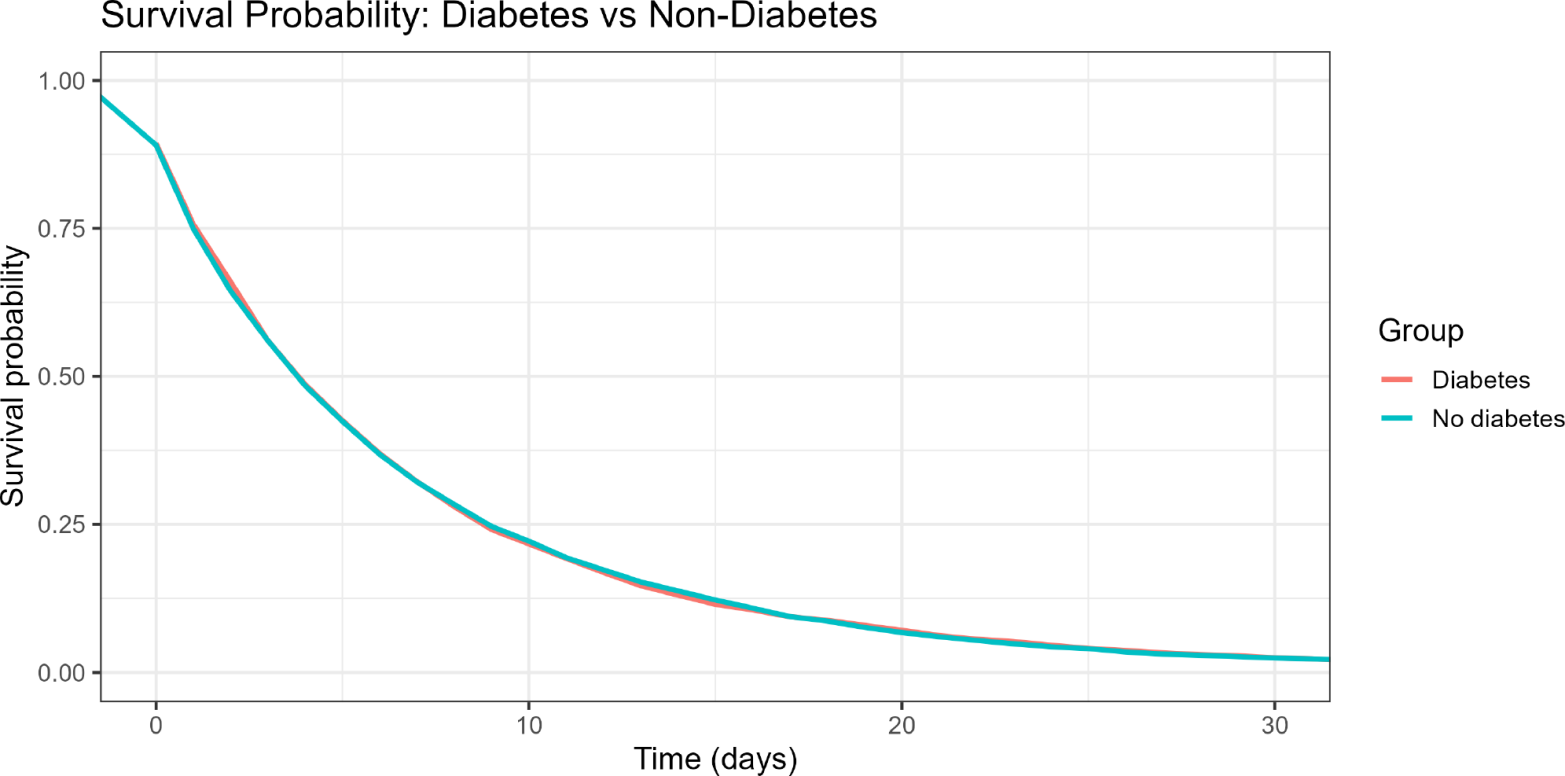
RMST univariable model for diabetic and non-diabetic patients.

RMST analyzes further quantified the impact of diabetes on short-term survival. **Table 5** shows that the difference in RMST between diabetic and non-diabetic patients over the chosen time horizon was approximately −0.19 (95% CI −0.282 to −0.0946), and the RMST ratio ranged from 0.89 to 0.90, indicating that diabetic patients experienced about 10% shorter mean survival than non-diabetic patients. In absolute terms, **Figure 4** shows that the RMST over 30 days was 6.36 days in the diabetic group and 8.23 days in the non-diabetic group, corresponding to an average loss of about 1.9 days of in-hospital survival associated with diabetes. Although the survival curves appear similar at each time point, small but persistent differences in survival probability accumulate and result in this clinically relevant RMST difference.

**Table 5:** Comparison of RMST between diabetes and no-diabetes groups.

Comparison	RMST estimate	95% confidence interval	*p*-value
Difference (Diabetes – No Diabetes)	−0.188	(−0.282, −0.095)	*<* 0.01
Ratio (Diabetes/No Diabetes)	0.90	(0.89, 0.91)	*<* 0.01

**Figure 5:**
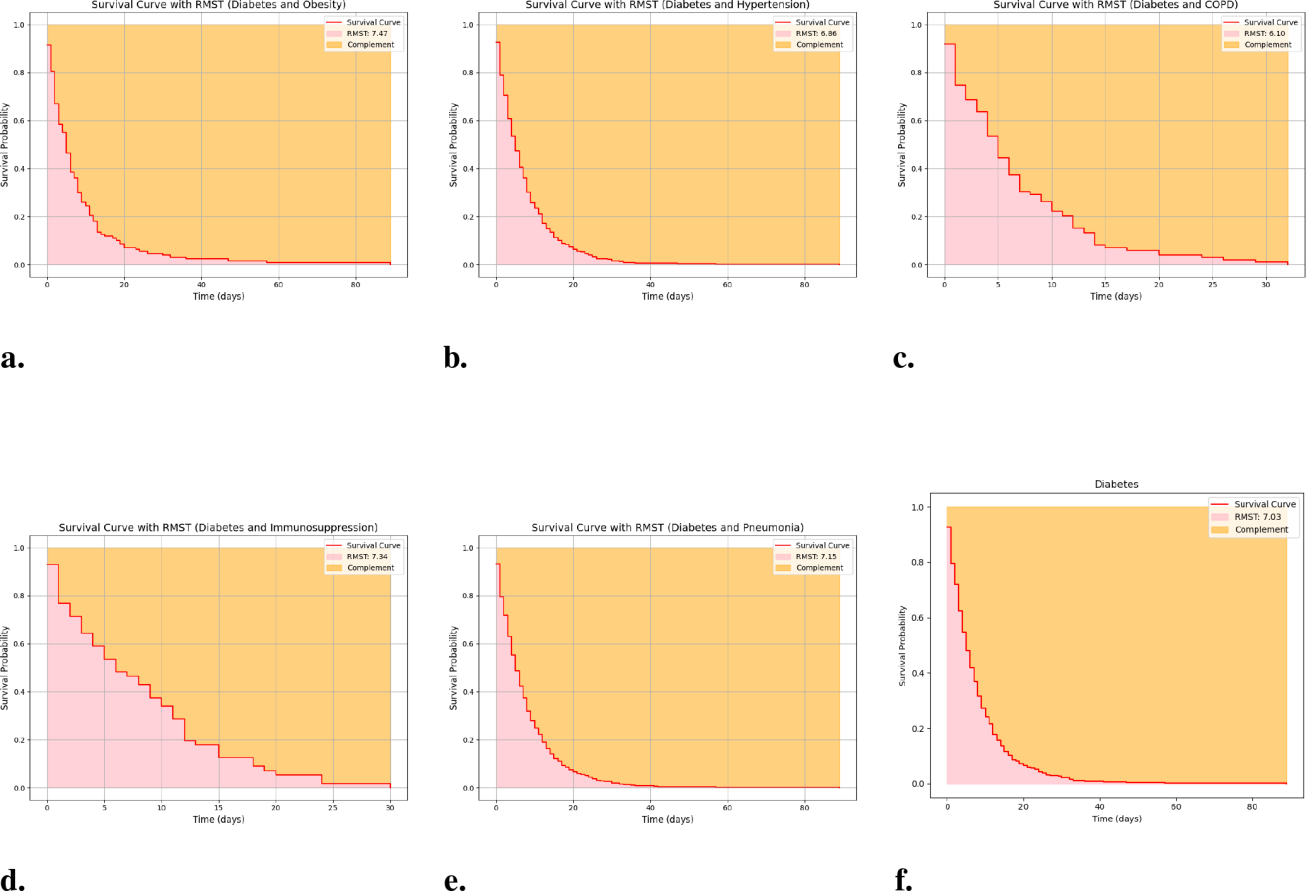
RMST multivariable models for diabetes and non-diabetes patients. **(A)** Diabetes + Obesity, **(B)** Diabetes + Hypertension, **(C)** Diabetes + COPD, **(D)** Diabetes + Immunosuppression, **(E)** Diabetes + Pneumonia, and **(F)** Diabetes + Smoking. **(A)**. RMST multivariable model over time for diabetes and obesity. **(B)**. RMST multivariable model over time for diabetes and hypertension. **(C)**. RMST multivariable model over time for diabetes and COPD. **(D)**. RMST multivariable model over time for diabetes and immunosuppression. **(E)**. RMST multivariable model over time for diabetes and pneumonia. **(F)**. RMST multivariable model over time for diabetes and smoking.

RMST analyzes stratified by comorbidity are presented in **Figure 5** and summarized in **Table 6**. Across time intervals, average RMST values for patients with diabetes combined with obesity, hypertension, immunosuppression, pneumonia, or smoking generally ranged from approximately 6 to 8 days, with notable temporal variability. Combinations such as diabetes with obesity or hypertension showed shorter RMST during mid-year periods, whereas diabetes with immunosuppression or pneumonia exhibited more pronounced fluctuations, suggesting that concomitant conditions and temporal factors jointly influence survival.

**Table 6:** Summary of RMST analysis across time intervals for various comorbidities.

Comorbidity	Min RMST (days)	Time of Min RMST	Max RMST (days)	Time of Max RMST	Avg. RMST (days)	Observations
Diabetes & Obesity	1.0	2023-06-30	12.05	2023-01-01	6.08	Shortest survival mid2023; longest early 2023.
Diabetes & Hypertension	3.63	2023-07-20	9.81	2023-05-21	6.55	Moderate variability; shorter survival mid-2023, longer in late spring.
Diabetes & COPD	0.0	2023-07-20	29.0	2023-06-30	8.21	High variability; wide temporal fluctuations in survival.
Diabetes & Immunosuppression	2.5	2023-05-21	12.0	2023-05-01	7.33	Moderate fluctuations; temporal patterns affecting survival.
Diabetes & Pneumonia	0.0	N/A	7.15	N/A	6.53	Strongly affected by pneumonia episodes.
Diabetes & Smoking	7.12	N/A	7.12	7.12	7.12	No consistent trend; highly variable survival.

**Figure 6:**
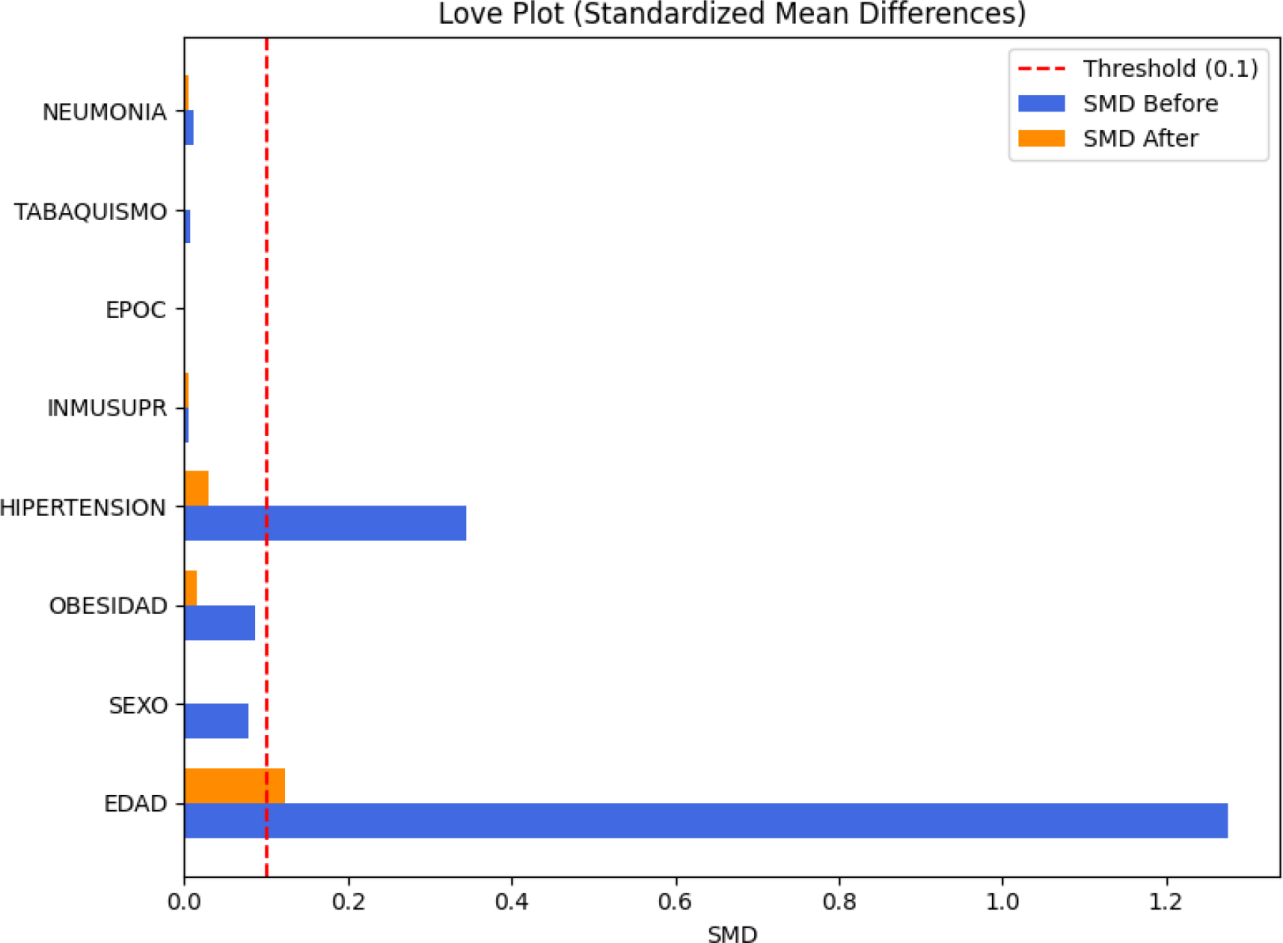
Standardized mean differences (SMD) before and after propensity score matching. All covariates achieved acceptable balance (*SMD<* 0.1), confirming that matching effectively minimized systematic differences between diabetic and non-diabetic groups.

To address potential confounding, we performed propensity score matching between patients with and without diabetes. After matching, the sample included 50,000 diabetic and 50,000 non-diabetic patients. Standardized mean differences for all covariates were reduced below 0.1, indicating adequate balance (**Figure 6**). In the matched cohort, RMST remained lower in the diabetic group, with a difference of 2.32 days; however, this did not reach conventional statistical significance (*p* = 0.0583). The magnitude of the difference nonetheless suggests a clinically meaningful trend toward shorter survival in diabetic patients, independent of major comorbidities.

### 3.2 Machine-learning prediction of RMST

**Figure 7:**
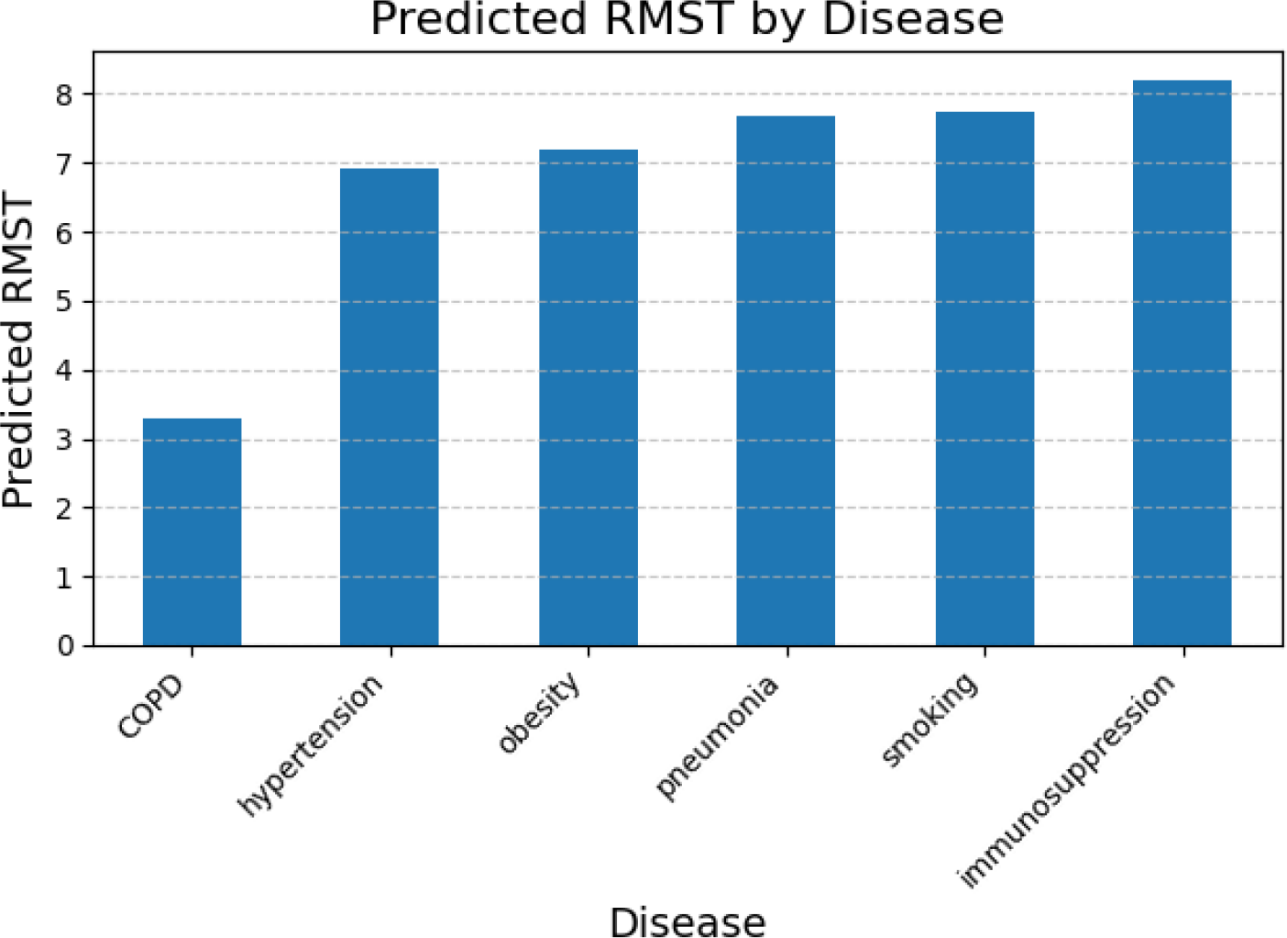
RMST predictions obtained using the random forest model. This plot highlights variations in RMST across different disease categories, illustrating the model’s ability to capture complex non-linear effects among comorbidities.

**Figure 8:**
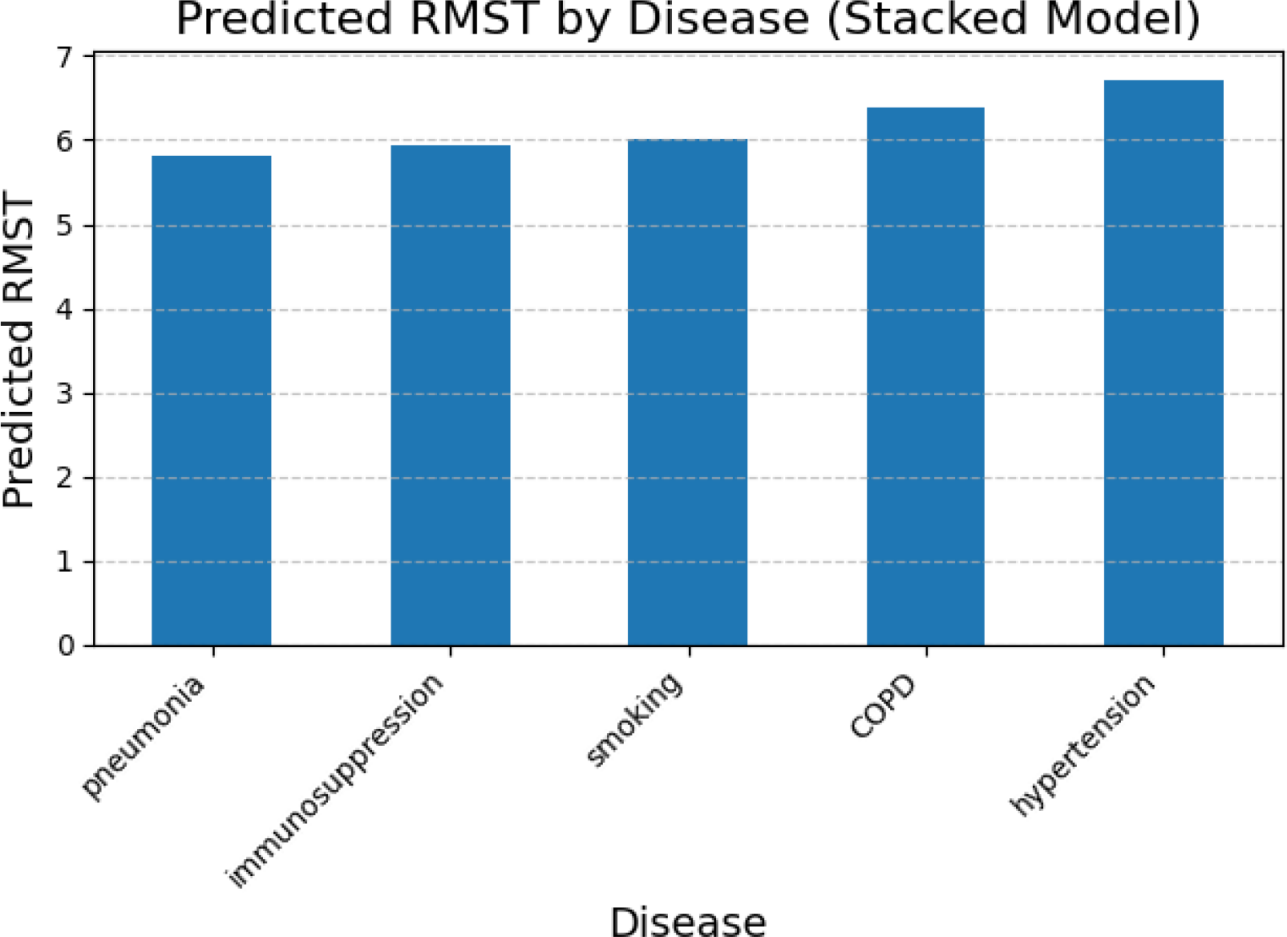
RMST predictions using the stacked model integrating RF and GBR. The figure highlights variations across different disease categories, showing that ensemble methods improve predictive accuracy and capture non-linear interactions between comorbidities.

We next evaluated machine-learning models for predicting RMST from aggregated survival summaries and comorbidity profiles. Models included RF, VDNN, and several stacked ensembles that combined tree-based and neural components. **Figures 7**, **8** illustrate predicted RMST values across disease categories.

**Figure 9:**
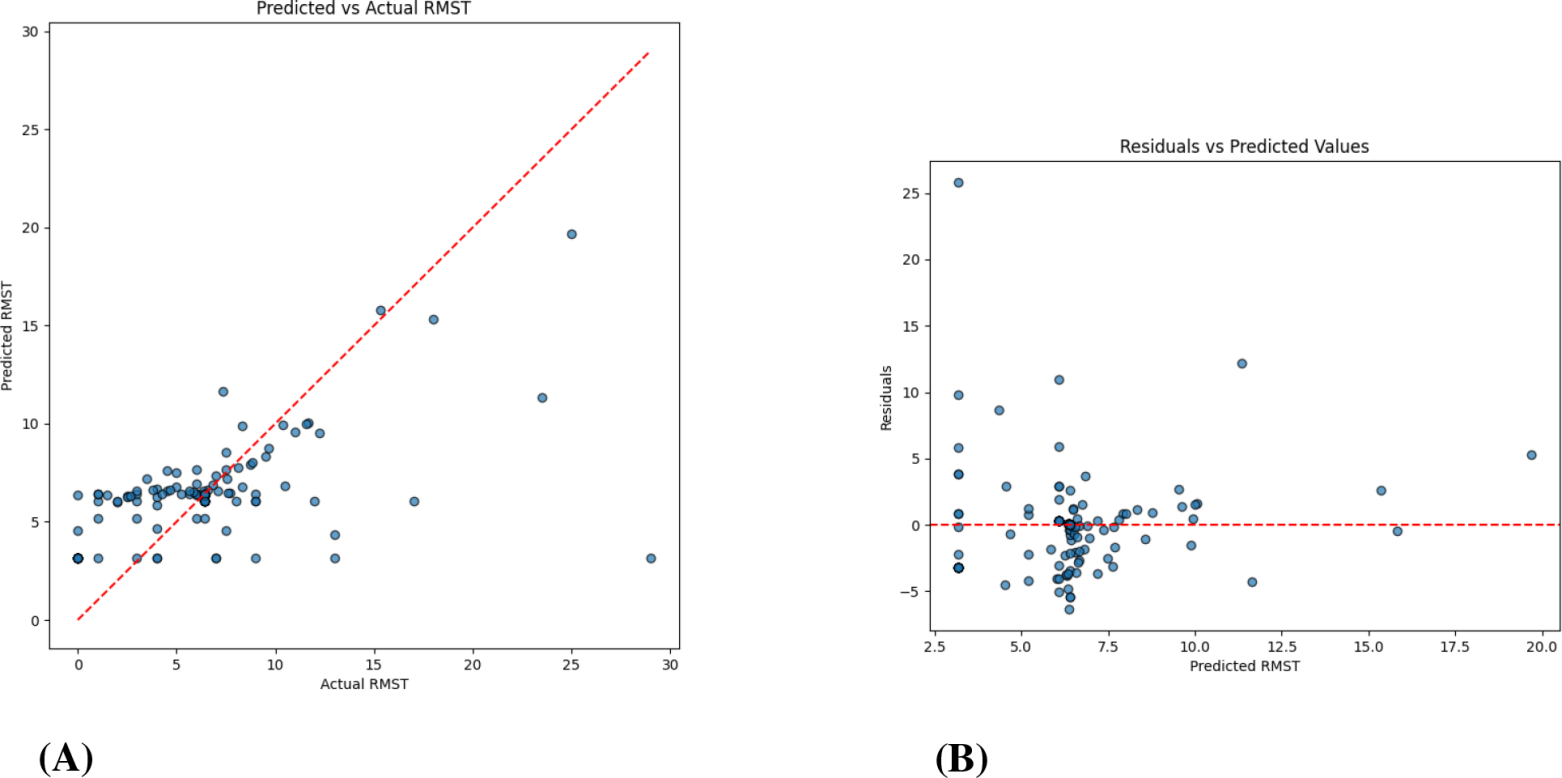
VDNN prediction performance. **(A)** Agreement between predicted and actual RMST values. **(B)** Residual analysis showing error distribution relative to predicted RMST values, confirming low bias and homoscedasticity. **(A)** Predicted vs. actual RMST values. **(B)** Residuals vs. predicted RMST values.

**Figure 10:**
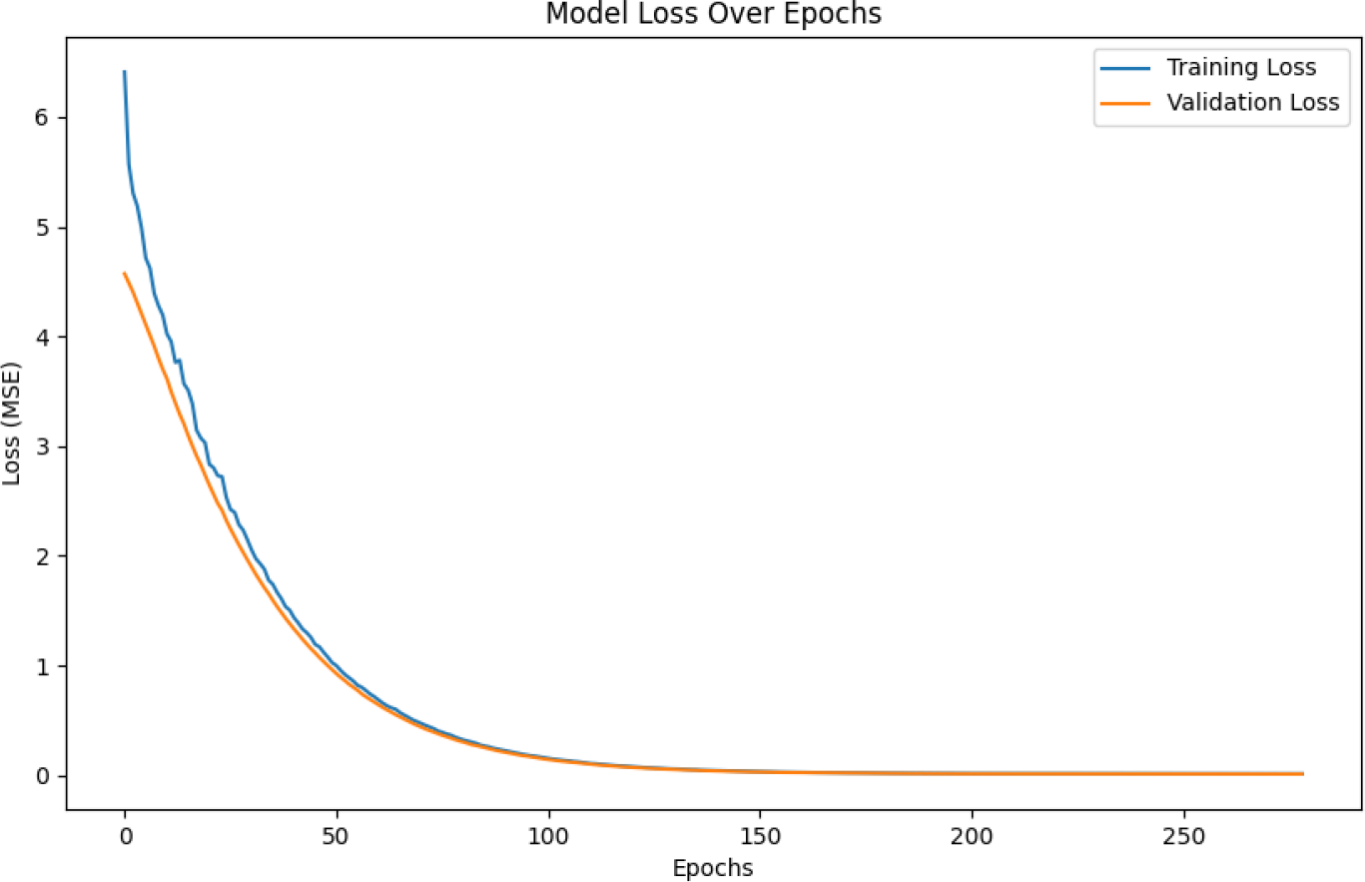
Model loss over training epochs for the Variational Deep Neural Network (VDNN). The plot illustrates convergence behavior and training stability, showing a consistent decrease in loss across epochs and confirming proper optimization of model parameters.

RF-based models captured clinically plausible patterns, with shorter predicted RMST for patients with COPD and longer values for those with pneumonia or immunosuppression, reflecting differences in disease severity and management. The VDNN provided a good overall fit but showed larger residual errors for extreme RMST values, with heteroscedasticity in the upper tail, as illustrated in **Figure 9** and the learning curves in **Figure 10**. These findings indicate challenges in accurately modeling rare, very long survival times.

**Table 7** compares model performance. The stacked ensemble combining RF and Gradient Boosting Regressor (GBR) achieved the best results, with the lowest mean squared error (MSE = 0.4699) and a near-perfect *R*^2^ of 0.9882, suggesting an excellent ability to reproduce observed RMST values. The RF alone had limited explanatory power (*R*^2^ = 0.0979), whereas the VDNN attained a moderate *R*^2^ of 0.35, indicating that it captured part, but not all, of the nonlinear structure in the data. The stacked model combining VDNN and RF performed similarly to the VDNN alone, highlighting the need for further optimization of this architecture.

**Table 7:** Comparison of predictive models for RMST prediction.

Model	MSE	*R* ^2^	Insights
RF	28.80	0.098	Moderate performance; limited variance explanation, suggesting need for tuning.
VDNN	14.11	0.35	Improved performance over RF, capturing nonlinear patterns more effectively.
Stacked Model (VDNN + RF)	14.06	0.33	Comparable to VDNN; minimal stacking benefit observed.
Stacked Model (RF + GBR)	0.47	0.988	Excellent performance; effectively integrates strengths of both methods.

All models were evaluated using an 80/20 train–test split and 5-fold cross-validation to assess generalizability. Ensemble approaches, particularly the RF+GBR stack, consistently outperformed single models, underscoring the benefits of combining complementary learners for RMST prediction in this clinical dataset. Nonetheless, model performance remains contingent on the quality and richness of the available features, and additional feature engineering and external validation will be required before implementation in clinical decision support.

## 4 Discussion

In this large retrospective national cohort of hospitalized adult COVID-19 patients in Mexico, we found that preexisting diabetes was associated with shorter survival times, with diabetic patients experiencing lower RMST than non-diabetic patients. The cohort showed a high burden of chronic conditions, with hypertension, diabetes, and obesity being particularly frequent, in line with previous reports from Mexico and other countries, where diabetes has been consistently linked to severe COVID-19 and adverse outcomes (20–24). The observed comorbidity patterns and mortality rates are comparable to those reported in Europe and other settings, further supporting the external consistency of our findings.

An apparent discrepancy arose between the multivariable Cox proportional hazards model and the Kaplan–Meier and RMST analyzes. While the Cox model yielded a non-significant hazard ratio for diabetes (HR ≈ 0.975, *p* = 0.167), KM curves and RMST estimates demonstrated significantly shorter survival among diabetic patients. This can be explained by several methodological factors. First, the Cox model adjusted for covariates that are strongly correlated with diabetes, such as hypertension and obesity, which may have introduced multicollinearity and attenuated the estimated independent effect of diabetes. Second, the proportional hazards assumption may only partially hold, whereas RMST does not rely on proportional hazards and summarizes average survival over a fixed time window, making it more robust in the presence of time-varying effects. Taken together, the combined use of Cox, KM, and RMST indicates that diabetes is clinically relevant for COVID-19 mortality even if its adjusted hazard ratio is attenuated in multivariable models.

The association between diabetes and poor COVID-19 outcomes is biologically plausible. Chronic inflammation, endothelial dysfunction, and impaired immune responses characteristic of diabetes increase susceptibility to respiratory infections and related complications (9, 10). The coexistence of diabetes with other cardiometabolic comorbidities further amplifies risk. Our results reinforce the need for targeted interventions in diabetic patients, including optimized glycemic control, early identification of clinical deterioration, and proactive management of co-occurring conditions (1, 25).

A major strength of this study is the use of a large, nationally representative dataset validated by the Mexican Ministry of Health, encompassing 475 monitoring units and including complete mortality information. The large sample size provided high statistical power and allowed detailed subgroup and propensity score matching analyzes. The quality of covariate balance achieved by the matching procedure is summarized in the Love plot shown in **Figure 6**, where standardized mean differences for all covariates fall below 0.1, indicating adequate post-matching balance. Nonetheless, several limitations must be acknowledged. The retrospective observational design precludes causal inference (26), and information on the duration and control of diabetes was unavailable. The absence of direct vaccination data limits our ability to disentangle the impact of immunization from other temporal changes in care. In addition, the findings may not be directly generalizable beyond the Mexican context due to differences in demographics, healthcare infrastructure, and epidemic dynamics, and the reliance on routine reporting introduces the possibility of misclassification and reporting bias.

**Figure 11:**
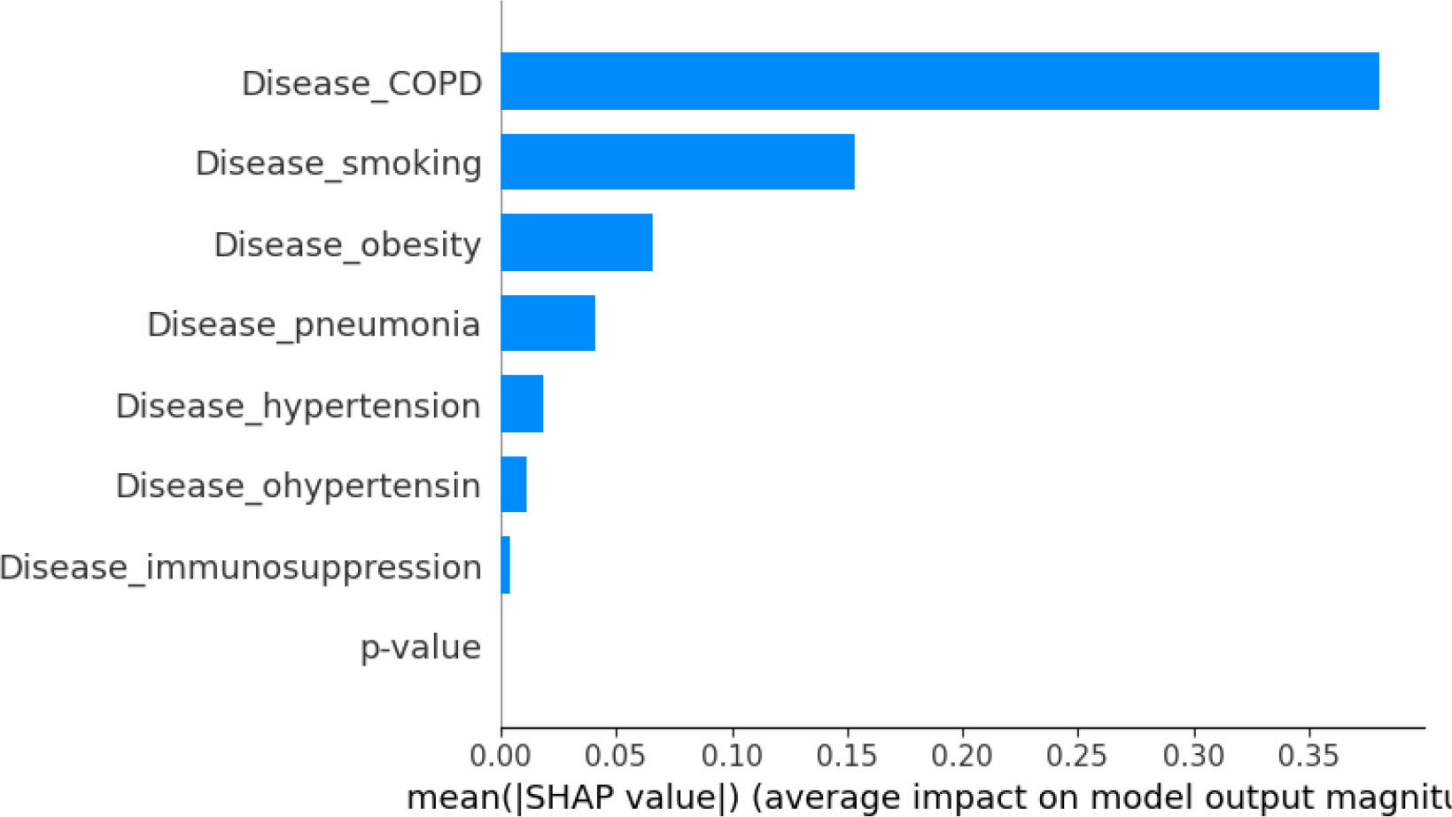
SHAP feature importance derived from the random forest model, illustrating the average impact of each input variable on RMST prediction. The model shows patterns consistent with the VDNN analysis, identifying comorbidities such as obesity and smoking, along with a late-period admission indicator, as among the most influential predictors of patient survival.

**Figure 12:**
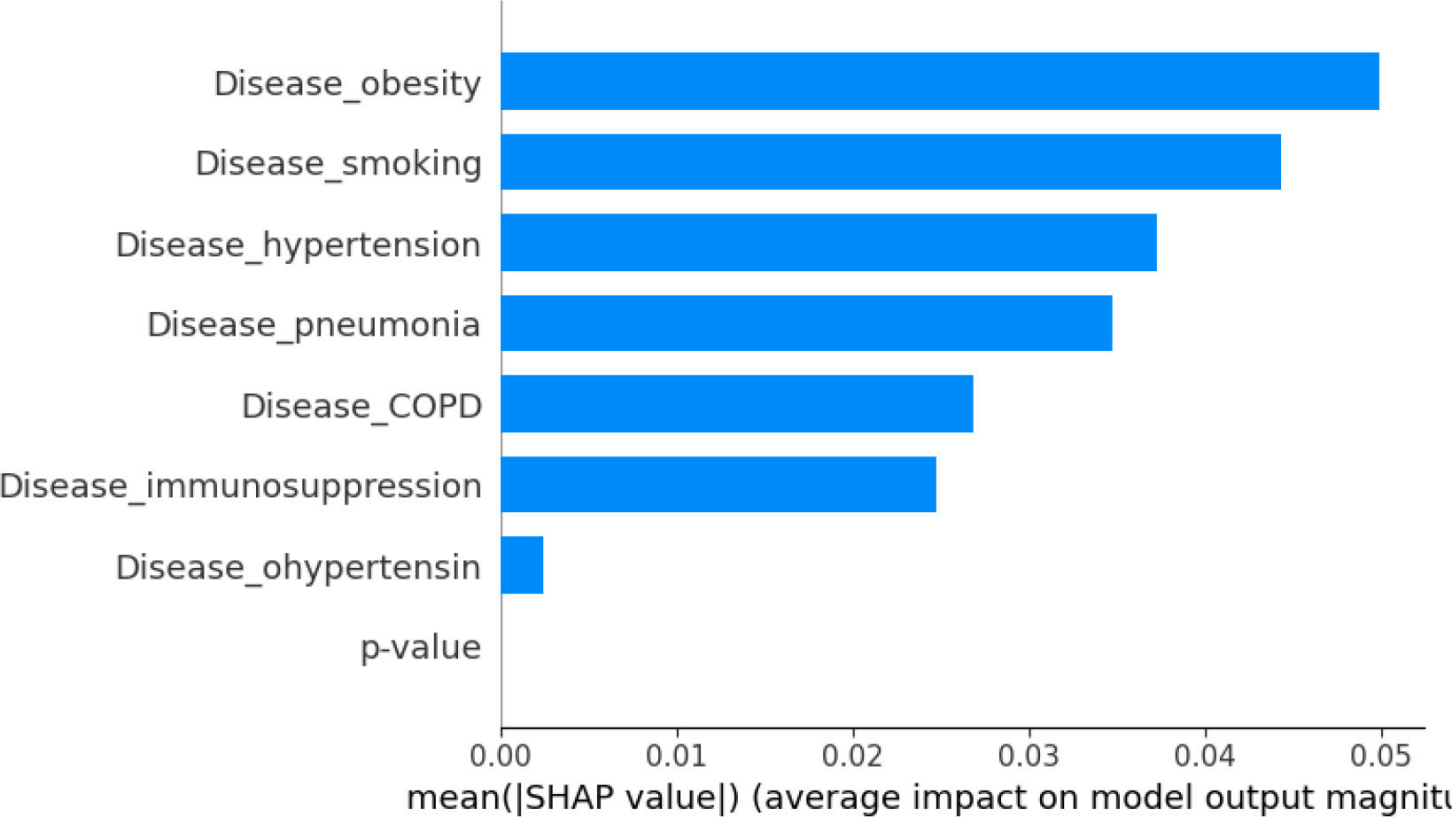
SHAP feature importance derived from the VDNN model, showing the average impact of each input variable on RMST prediction. Key contributors include comorbidities such as obesity and smoking, as well as a binary temporal indicator corresponding to post-vaccination admissions (after March 2023).

**Figure 13:**
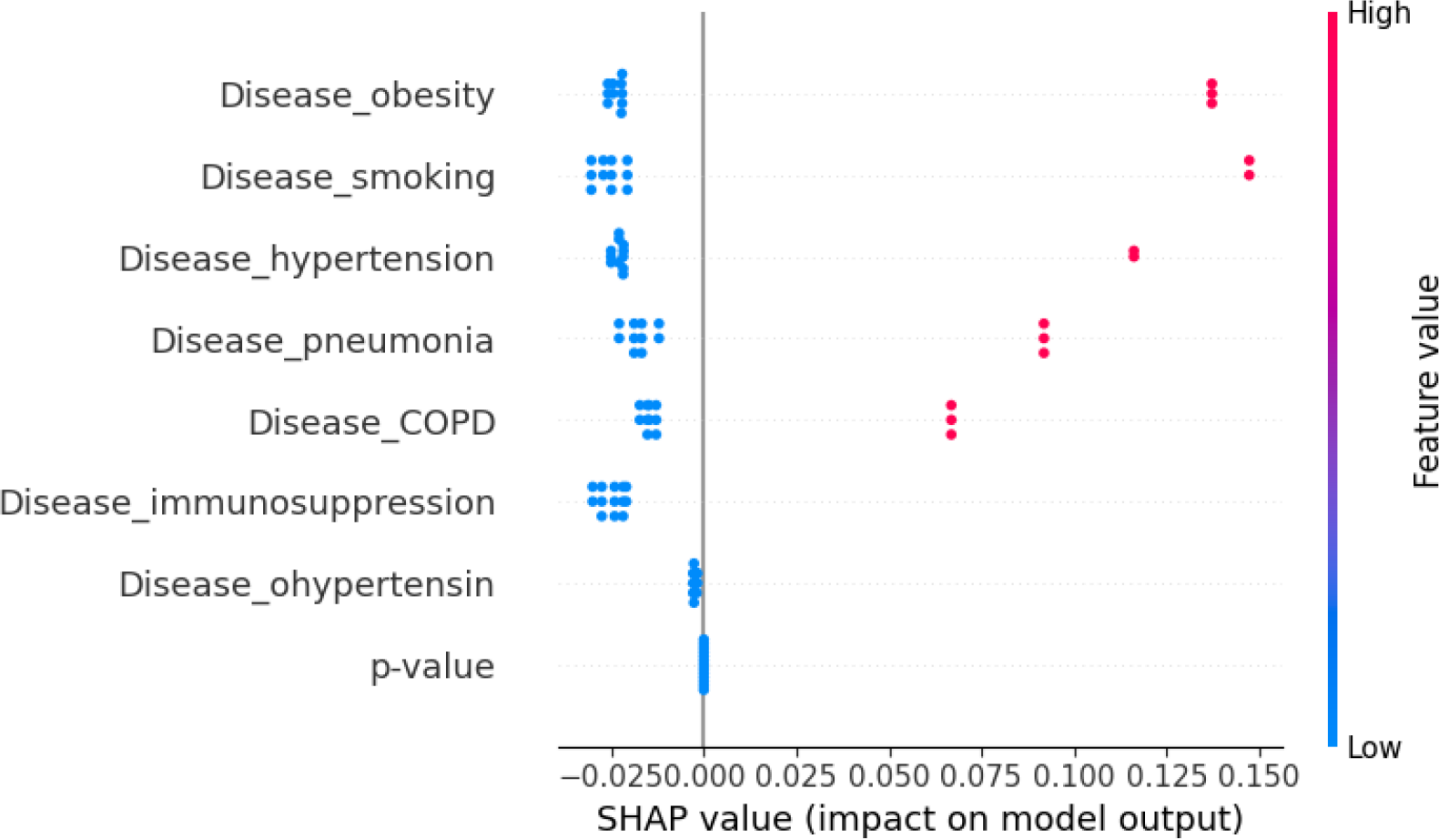
SHAP summary plot for the VDNN model. Each dot represents an individual prediction, with color indicating the corresponding feature value (red = higher values, blue = lower values). Features such as obesity and late-period admission are associated with increased RMST predictions, reflecting their significant contribution to survival outcomes.

**Figure 14:**
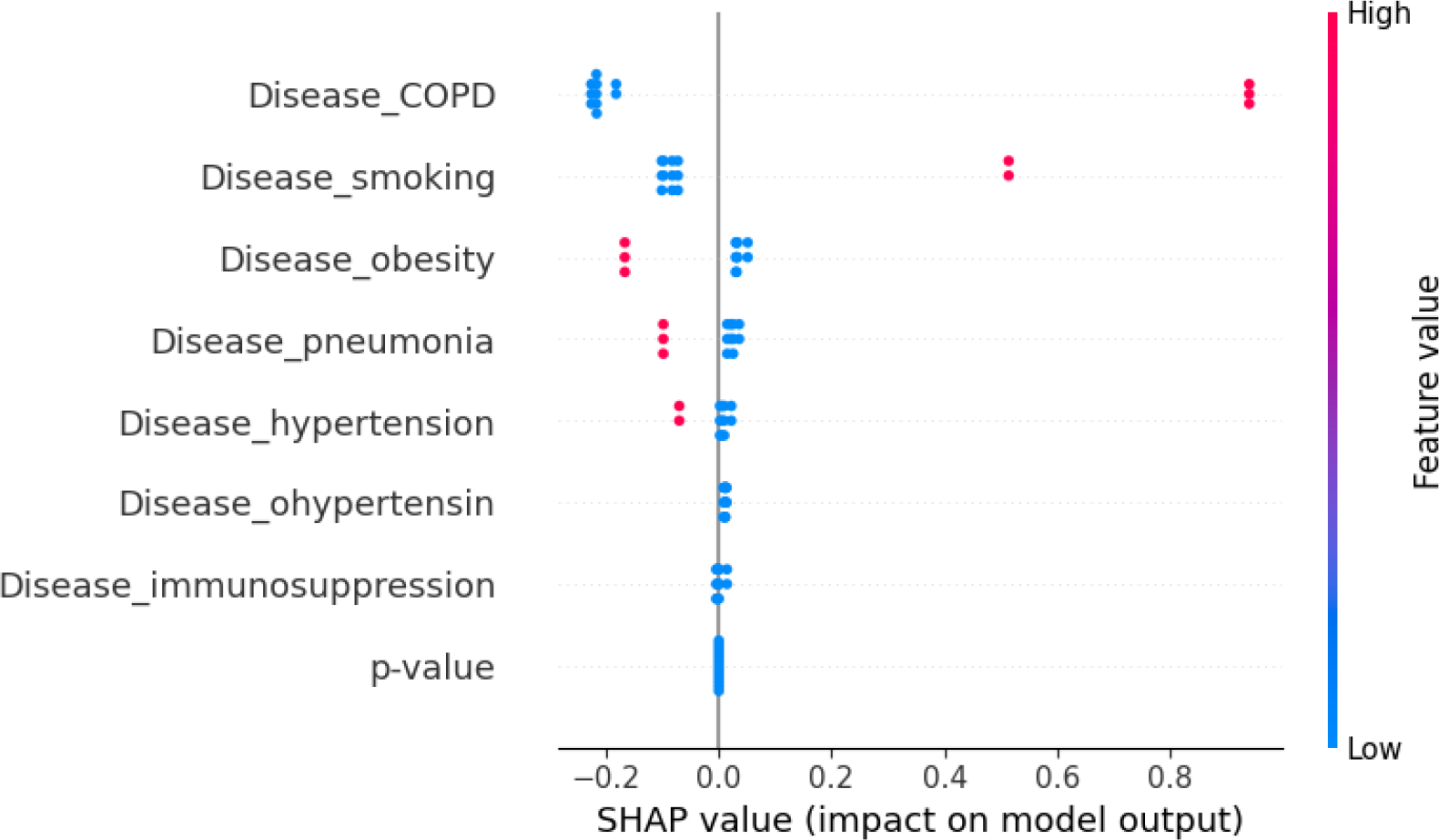
SHAP summary plot for the random forest model. Each point represents a patient instance, colored by the feature value, indicating how individual predictors influence the RMST estimate. Feature effects are largely consistent with those obtained from the VDNN model, confirming robustness of the learned importance structure across architectures.

We also evaluated machine-learning models to predict RMST from comorbidity and temporal profiles. RF and VDNN models captured nonlinear relationships between covariates and RMST, and their combination in stacked ensembles further improved predictive performance. The RF+GBR stacking model achieved the best internal performance, highlighting the potential of ensemble methods for short-term survival prediction in large clinical datasets. Using SHAP values, as summarized in the feature-importance bar plots in **Figures 11**, **12**, we identified obesity, smoking, hypertension, and a temporal indicator (PostVaccine, defined as admission after March 1, 2023) as key contributors to predicted RMST. The SHAP summary plots for the VDNN and Random Forest models (**Figures 13**, **14**) further illustrate that these features consistently exert a substantial influence on the predicted survival time across individual strata, confirming the robustness of their effects across different architectures. The overall data transformation pipeline from raw patient-level records to the aggregated RMST-based machine-learning dataset used for these models is summarized in **Figure 1**.

Traditional survival analysis and machine learning provide complementary perspectives on patient risk. Classical methods such as the Cox proportional hazards model and RMST are primarily designed for population-level inference, quantifying effect sizes and differences in average survival between clinical groups. In contrast, our machine learning models operate on RMST estimates computed for predefined comorbidity–time strata, capturing nonlinear relationships and interactions among covariates that are difficult to model parametrically. In this way, inferential survival tools quantify the impact of diabetes on group-level survival, while ensemble and neural models enhance the prediction of expected short-term survival for specific risk profiles. This dual framework allows us to link rigorous statistical association measures with improved predictive performance, thereby supporting more nuanced, data-driven risk stratification.

Although our models showed strong internal validity under cross-validation, external validation in independent datasets was not performed and remains an important next step, particularly in other low- and middle-income country settings. Future work should focus on refining model architectures, incorporating additional clinically relevant features, and validating predictive performance and calibration across diverse populations. At the same time, survival analysis and machine learning should be viewed as complementary: classical methods quantify group-level effects and provide interpretable effect estimates, whereas machine learning models offer flexible tools for predicting short-term survival for specific risk profiles and exploring complex patterns in high-dimensional data.

## 5 Conclusion

In this national cohort of hospitalized adult COVID-19 patients in Mexico, diabetes was associated with shorter survival times, as reflected by lower RMST estimates compared with non-diabetic patients. These findings reinforce the heightened vulnerability of individuals with diabetes to severe COVID-19 outcomes and underscore the need for targeted clinical management and prioritized allocation of healthcare resources in this subgroup.

By combining traditional survival analysis with machine-learning models, we quantified both group-level differences in survival and the contribution of comorbidities and temporal factors to predicted short-term survival. Ensemble approaches, particularly stacked models integrating tree-based and neural predictors, achieved high predictive accuracy and illustrate the potential of hybrid analytic frameworks for risk stratification in large clinical datasets. Future work should prioritize the external validation of our model in other low- and middle-income country settings to assess its transferability and identify population-specific modifications needed to optimize survival prediction accuracy. Such validation across geographically and demographically distinct populations, each with unique comorbidity profiles, viral circulation patterns, and healthcare constraints, will be essential to determine the broader applicability and robustness of the proposed framework.

## Data Availability

The original contributions presented in the study are included in the article/supplementary material. Further inquiries can be directed to the corresponding author.

## Appendix

**Table 8** summarizes the baseline characteristics of hospitalized COVID-19 patients according to diabetes status. Among 1,021,380 hospitalized individuals, 52,257 (5.1%) had diabetes, and 969,123 (94.9%) did not. Patients with diabetes were notably older than those without diabetes, and the proportion of male patients was similar in both groups, with a slightly higher percentage among individuals with diabetes (53.7% vs. 51.8%).

**Table 8:** Summary of baseline characteristics comparing diabetic and non-diabetic patients hospitalized with COVID-19.

Characteristic	Non-diabetic (N = 969,123)	Diabetic (N = 52,257)
Age, mean (SD)	49.5 (17.2)	61.8 (12.3)
Age ≥ 60 years, n (%)	315,268 (32.5%)	35,487 (67.9%)
Male, n (%)	502,221 (51.8%)	28,087 (53.7%)
Female, n (%)	466,902 (48.2%)	24,170 (46.3%)
Comorbidities		
Hypertension, n (%)	140,522 (14.5%)	32,764 (62.7%)
Obesity, n (%)	129,812 (13.4%)	16,437 (31.4%)
COPD, n (%)	16,482 (1.7%)	2,481 (4.7%)
Immunosuppression, n (%)	7,904 (0.8%)	1,265 (2.4%)
Smoking, n (%)	74,101 (7.6%)	6,329 (12.1%)
Other hypertension (oHT), n (%)	3,381 (0.3%)	554 (1.1%)

As expected, cardiometabolic and respiratory comorbidities were substantially more prevalent in the diabetic group. Hypertension affected nearly two thirds of diabetic patients (62.7%) compared with only 14.5% of non-diabetic patients, and obesity was more than twice as frequent among those with diabetes (31.4% vs. 13.4%). Likewise, smoking (12.1% vs. 7.6%) and other hypertension (1.1% vs. 0.3%) were more common in patients with diabetes than in those without. Overall, these findings indicate that hospitalized COVID-19 patients with diabetes constitute an older and more comorbid subgroup, with a marked clustering of hypertension, obesity, and other high-risk conditions.
